# Teleportation in Virtual Reality; A Mini-Review

**DOI:** 10.3389/frvir.2021.730792

**Published:** 2021-10-18

**Authors:** Aniruddha Prithul, Isayas Berhe Adhanom, Eelke Folmer

**Affiliations:** Department of Computer Science and Engineering, University of Nevada, Reno, NV, United States

**Keywords:** virtual realty, virtual locomotion, teleportation, VR sickness, navigation

## Abstract

Teleportation is a widely implemented virtual locomotion technique that allows users to navigate beyond the confines of available tracking space with a low possibility of inducing virtual reality (VR) sickness. This paper provides a comprehensive overview of prior research on teleportation. We report results from user studies that have evaluated teleportation in comparison to other locomotion methods and survey improved versions of teleportation. We identify a number of areas for future research.

## 1 INTRODUCTION

One of the major challenges VR developers and researchers face is the problem of virtual locomotion (Al Zayer et al., 2020), i.e., techniques that allow a user to navigate efficiently in a virtual environment without getting VR sick. To maximize presence, an ideal locomotion technique would map one’s movement one-to-one with their virtual avatar. In most consumer VR systems, to some extent this can be achieved with room scale tracking. However, such tracked spaces are finite in size and restricted by available physical space. To travel beyond these confines, most VR applications need to implement a Virtual Locomotion Technique (VLT).

First mentioned by Mine (1995), teleportation is a common VLT used to traverse larger spaces. The user may specify a destination in the virtual environment (VE) by pointing. This is commonly done using a handheld motion tracked controller. When activated, the user’s viewpoint may instantly transition to the specified destination. A benefit of this instant viewpoint transition is that it does not generate any optical flow. Optical flow cues that simulate self-translation or self-rotation create an illusion of self-motion called vection, which, in the absence of any vestibular/proprioceptive afferents, can confuse the senses and lead to vection-induced VR sickness (Bonato et al., 2009). Because VR sickness is a major concern for VR, teleportation has been widely implemented as a “safe” VLT with a low risk of inducing VR sickness.

This paper provides a comprehensive overview of studies that have compared the different types of teleportation to other VLTs, as well as an overview of improved versions of teleportation.

## 2 SURVEY METHODOLOGY

Our aim with this review is to examine the breadth, range and nature of research activities regarding the use of teleportation in VR. To identify all relevant studies, we used the snowballing search method (Wohlin, 2014). The snowballing search method identifies a set of initial papers and uses the reference list of these papers (backward snowballing) or the citations to these papers (forward snowballing) to search for relevant studies (Wohlin, 2014). For this survey, we used the following inclusion criteria:

- Peer reviewed papers, i.e., those published as a journal, conference paper or as a dissertation.
- Papers that included a study where teleportation was evaluated empirically with users.
- Papers that used an HMD for VR. Given that HMDs are currently the most widely adopted VR platform, we excluded papers that used older VR technology like CAVEs or large screens.

We employed the snowballing approach by starting with two well-referenced studies as our initial set (Bowman et al., 1997; Bozgeyikli et al., 2016). Article selection was made according to the previously stated inclusion criteria. We, finally, selected 27 papers to include in our survey, including the initial two papers. The bibliographic management software Mendeley was used to organize the articles. An analysis of all found papers revealed these could be classified into two categories: 1) Studies with teleportation in comparison to other VLTs (surveyed in Section 3); and 2) Improvements of teleportation (surveyed in Section 4).

## 3 STUDIES WITH TELEPORTATION

Teleportation has become the *de facto* locomotion method for many VR experiences and is often used as a benchmark to compare new VLTs against. We can classify the different teleportation implementations used in the surveyed papers based on a few distinguishing features:

- **Operation:** The user uses a motion sensing hand held controller to point towards a destination to teleport to and then activates teleport using a button press. Alternatively, handsfree approaches for selecting a destination are possible using eye gaze, head gaze or by pointing with the arm or hand which are tracked externally using a camera.
- **Translation:** When teleport is activated the user’s avatar is instantly translated to the destination (discontinuous translation) or alternatively the user’s avatar is moved rapidly maintaining a continuous representation.
- **Concordance:** A few implementations allow users to specify their orientation post-teleport (discordant) or they maintain their orientation (partially concordant).
- **Range:** Users can teleport to any destination within their visible range (commonly within a specific distance threshold). Contrarily, they may only be allowed to teleport to set locations in the environment.

This section summarizes significant findings (presented in chronological order) as they pertain to teleportation. User study details, such as the type of task used in the study and number of participants can be found in Table 1.

Bowman et al. (1997) compared gaze directed and pointing based locomotion methods with both continuous and instant viewpoint change conditions. In the absolute motion experiment, which closely resembles teleportation, no significant differences were found between the two teleportation-like techniques. On the other hand, compared to continuous viewpoint transition, instant viewpoint transition (teleportation) was found to cause more spatial disorientation.

Bakker et al. (2003) Compared teleportation with and without anticipation. They found that anticipation to an extent helped avoiding disorientation resulting from teleportation. When teleportation was accompanied by viewpoint change (rotation of the viewpoint), the anticipation time required to avoid disorientation increased.

The majority of studies have compared teleportation to other VLTs. Christou and Aristidou (2017) compared teleportation to gaze directed and pointing based steering VLTs. In their finding, teleportation had lower cybersickness compared to gaze and pointing directed steering. While teleportation was faster than steering, there was a tendency to miss environmental details when teleporting. The study didn’t find any drawbacks to teleportation regarding spatial disorientation when compared to steering.

Frommel et al. (2017) compared four controller based VLTs-fixed point teleport, free teleport, joystick and automatic locomotion. Free teleport was found to cause lower VR sickness and high presence as well as enjoyment factor.

Lindal et al. (2018) compared free teleport with fixed path navigation (user only controls speed). Simulator sickness increased for both VLTs with no significant difference between them. While the positive association towards VR decreased for fixed path navigation, it remained the same for teleportation. Heart rate was significantly higher for teleportation.

Vlahovic et al. (2018) compared teleportation to three continuous VLTs, i.e., joystick, joystick with tunneling (where the field of view was restricted), and body tilt. There was no difference in presence, but quality of experience was significantly higher for teleportation.

Langbehn et al. (2018) compared joystick, teleportation and redirected walking (RDW). Cognitive map building was tested using a pointing and a spatial arrangement task. Travel time was shortest for teleportation and joystick had higher VR sickness than RDW and teleportation. Teleportation and joystick had worse cognitive mapping than RDW. There was no difference in presence scores (which were low) between the three VLTs, but teleportation and RDW were most preferred.

Coomer et al. (2018) compared arm cycling, joystick, point-tugging (users select a point in space and pull themselves toward it by pushing on a button on a controller) - which were all continuous VLTs, to teleportation. Their results found that arm cycling was superior to teleportation in terms of enjoyment and task completion time. Teleportation and arm-cycling had lower simulator sickness than joystick and point-tugging. Users travelled significantly further using teleportation and users looked around more as teleportation caused more spatial disorientation than the other VLTS. Teleportation caused less fatigue compared to arm cycling.

Griffin et al. (2018) compared two hands-free VLTs: head-tilt and walking-in-place (WIP) to two controller based VLTs: joystick and teleportation using a bimanual target shooting while navigating tasks. Teleportation had the lowest navigation time while also having the lowest VR sickness incidence. Compared to WIP and head-tilt, teleportation had the lowest presence and also a lower cognitive load than WIP.

Clifton and Palmisano (2019) compared steering (i.e., pointing the controller in the direction of travel with a button press activating travel) to teleportation. A user study with seated and standing conditions found that teleportation had a lower cybersickness incidence and presence than steering.

Bozgeyikli et al. (2019) compared redirected walking, walk-in-place, stepper machine, teleport, joystick, trackball, hand flapping and flying with and without obstacles. with and without obstacles. Teleportation resulted in the fastest locomotion time without obstacles and the lowest number of collisions with obstacles. Participants also ranked teleport the highest from all tested VLTs.

Loup and Loup-Escande (2019) compared arm swinging to teleportation. There was no difference in completion time. Teleportation had significantly lower cybersickness incidence, a lower cognitive load, and a better user experience.

Paris et al. (2019) compared two discrete VLTs, i.e., teleportation and point-tugging to two continuous VLTs, i.e., skiing and flying. The user study used a triangle completion task which is a well known task to assess path integration ability Bhandari et al. (2018a). This task involves users following two legs of a triangle, after which they would have to point towards their starting location. Discrete and continuous VLTs were pooled together. No differences were found between continuous and discrete VLTs regarding simulator sickness or presence. Discrete VLTs (especially teleportation) had a higher path integration error and thus were more likely to lead to spatial disorientation.

Boletsis and Cedergren (2019) compared WIP, joystick, and teleportation. Teleportation was most efficient, but less immersive than WIP.

Drogemuller et al. (2020) compared teleportation, one handed flying, two handed flying and world-in-miniature (Stoakley et al., 1995) (i.e., a popup map that allows users to select a destination to be teleported to) in a 3D graph environment. They found teleportation to be the least suitable technique for this specific type of environment. Teleportation was found to be the slowest and least preferred.

Cherep et al. (2020) compared walking, partially-concordant teleportation and discordant teleportation, i.e., Point and Teleport (Bozgeyikli et al., 2016). Spatial updating performance was best for walking and worst for point and teleport with partially-concordant teleport falling in between. These results emphasize the importance of transnational and rotational self-motion cues in spatial navigation performance. The study was extended upon by Kelly et al. (2020) where they also measured the influence of path scale and found the importance of rotational self-motion cue was exaggerated with larger path scale.

### 3.1 Summary

Table 1 summarizes how teleportation affects particular factors from each of the studies surveyed in the previous section. We classify each type of teleportation, user study details and what VLTs the proposed technique was compared to. We then include factors that we have extracted from the outcome of the studies. Factors were only included if at least three studies found a significant effect (positive or negative) and therefore certain reported factors like heart rate, cognitive load and positional tracking usage were not included in the table. We took the liberty to group certain factors together, i.e., speed and efficiency under performance; cybersickness and simulator sickness under VR sickness; and user experience and quality of experience under usability. We also merged immersion with presence, though we are aware that those are closely related yet different.

## 4 TELEPORTATION IMPROVEMENTS

This section surveys improved versions of teleportation and papers are discussed in chronological order.

The “Jumper Metaphor” by Bolte et al. (2011) is a handsfree version of teleportation. The user selects a destination by looking at it for 500 milliseconds. The jump is initiated when the user moves towards the target with acceleration higher than a threshold value. Jumper was found to be superior to regular teleportation in terms of spatial disorientation, satisfaction and ease of use.

Bozgeyikli et al. (2016) presents “Point and Teleport”. Users select a destination by pointing using their index finger that is tracked using an external camera and by holding this pose for 2 s it activates teleport. A novel feature is that the user can specify their avatar’s orientation post-teleport, which minimizes the need for a user to correct their orientation after teleporting. A reticle with an embedded arrow shows the user’s post-teleport orientation which can be modified by rotating the hand used for pointing. Compared to regular teleport no difference in travel time or effort was found.

Linn (2017) is a handsfree version of teleportation that lets users select a destination to teleport using eye gaze. A user study found no differences in efficiency or presence with controller based teleportation though gaze based teleport was preferred.

Bhandari et al. (2018b) presents a teleportation method called ‘Dash’ that aims to reduce spatial disorientation by providing a small amount of optical flow during the viewpoint transition to allow for path integration. Compared to regular teleportation, Dash enables path integration and reduces spatial disorientation without increasing VR sickness.

Liu et al. (2018) presents redirected teleportation; a version of teleportation that uses iterative reorientation and re-positioning using a portal to non-obtrusively redirect the user back to the center of the tracking space, where available walking space is larger. Redirected teleport required significantly fewer teleports than regular teleport with users walking more and using more of the available tracking space.

Two techniques have explored offering a third person perspective to VR which is typically experienced from a first person perspective. Outstanding by Cmentowski et al. (2019) and out-of-body locomotion by Griffin and Folmer (2019) let users switch between first and third person perspective with the press of a button. While outstanding lets users navigate their avatar over long distances using raycast aiming from a birds eye view, out-of-body locomotion uses the touchpad. Upon breaking line of sight out-of-body locomotion switches back to first person perspective. User studies found Outstanding to increase spatial awareness due to the elevated perspective, while out-of-body locomotion found a significant reduction in the number of teleports required compared to regular teleport with no differences for other factors like performance or VR sickness.

Funk et al. (2019) evaluated two novel improvements of point and teleport (Bozgeyikli et al., 2016) for selecting a destination and specifying the avatar’s orientation post teleport. ‘Curved Teleport’ uses a parabolic curve which can be rotated over the horizontal plane by changing the roll of the controller and lets users teleport around an obstacle. “HighPointCurved” Teleport combines parabolic and curved teleport. Both techniques increase travel time but reduce the need for the user to correct their orientation after teleportation, which increases usability.

Von Willich et al. (2020) presents “Podoportation” which is another handsfree teleportation technique that lets users specify a teleport destination using their feet. Shoes were instrumented with sensors and nine different foot input techniques were evaluated versus controller based teleportation. A user study demonstrated its feasibility though performance and selection accuracy were lower than using a conventional controller.

Weissker et al. (2019) presents a group based teleportation technique named Multi-Ray Jumping that allows two users to navigate a VE together. It was found to improve planning accuracy (performance), reduce cognitive load and lower disorientation for passengers.

### 4.1 Summary

Table 2 summarizes the surveyed teleportation improvements. We classify each type of teleportation, user study details and what VLTs the technique was compared to in the same way as Table 1. We then categorize each improvement using five of the factors (performance, presence, spatial disorientation, and usability) that were identified in Table 1 and a sixth factor, Handsfree, for papers that proposed handsfree versions of teleportation.

## 5 DISCUSSION AND FUTURE WORK

The majority of studies with teleportation [except Lindal et al. (2018)] found that teleportation causes little to no VR sickness, which confirms a desirable feature about teleportation. Most studies [except Drogemuller et al. (2020) for navigating 3D graphs] found teleportation to offer a higher performance than other VLTs, but it comes at a cost of a lower presence and an increase in spatial disorientation. These effects are explained by teleportation’s instant viewpoint transition which allows instant travel. The lack of optical flow mitigates vection induced VR sickness but also limits path integration which increases spatial disorientation.

Most studies have primarily used a navigation or search task in user studies that compare VLTs. Given that teleportation relies on using a controller, this is a limitation as in popular VR experiences like games, controllers are already used for various other time critical tasks. For example, shooting at enemies or interacting with objects. So far only one study Griffin et al. (2018) has evaluated teleportation and other VLTs under such specific conditions. Future studies that aim to evaluate teleportation should include such more realistic usage scenarios.

Regarding teleportation improvements, few papers specifically set out to improve a known disadvantage of teleportation, i.e., Bhandari et al. (2018b) specifically aimed to reduce spatial disorientation whereas others [i.e., Bolte et al. (2011) and Cmentowski et al. (2019)] found spatial awareness to increase as a side effect. Liu et al. (2018) set out to improve tracking space utilization, but this was not a previously identified problem with teleportation. Tracking space utilization of various VLTs and especially teleportation would be interesting to investigate as available tracking space in home environments is often limited.

Because teleportation doesn’t resemble any real world form of travel, it is considered to offer a low presence. Only one paper (Liu et al., 2018) claims their teleportation technique improves presence because it requires fewer teleports and enables more walking which has the highest presence. However, this is an indirect claim that has not been verified with any users. Many studies have not even attempted to evaluate presence given that teleportation is considered to have a low presence anyway. VR holds significant promise to transform social interaction. Though multi-user teleportation mechanisms have been explored (Weibker et al., 2018), how teleportation is perceived by other users and most importantly how it affects presence or gameplay has not been the focus of any studies. There is some anecdotal evidence (Sumner, 2017) that the use of teleportation in multi-user environments is problematic as the discontinuous representation of a user’s avatars makes it impossible to follow or predict the path of a user, which is required to play multiplayer games like first person shooters. Randomly appearing and disappearing avatars is likely also to be detrimental to presence. Using a third person perspective Cmentowski et al. (2019); Griffin and Folmer (2019) which maintains a continuous avatar representation could be a solution but how it affects presence has not been studied in multi-user environments yet. With teleportation likely continuing being the default VLT for many VR experiences, figuring out how its presence can be improved, especially in multi-user environments, would be an important question for future research.

Several papers evaluated the usability or performance of an improved version of teleportation in comparison with regular teleportation but did not find a significant difference. This does not mean both techniques were equivalent to each other especially as the number of participants in many of these user studies were quite low. It is interesting to note that some teleportation improvements can already be found in commercially available VR games, e.g., post teleport orientation specification (Bozgeyikli et al., 2016) can be found in the VR shooter Robo Recall, while both Raw Data and Half-Life Alyx feature a dash-like (Bhandari et al., 2018b) viewpoint transition.

## 6 CONCLUSION

This mini review surveys studies comparing teleportation to other VLTs and improvements of teleportation. We categorize studies based on what type of teleport they use and how they improve certain factors like presence, performance, disorientation and VR sickness. We also identify several areas for future research.

## Figures and Tables

**TABLE 1 |:** The table summarizes how teleportation affects certain factors based on the surveyed studies.

	Bowman et al. (1997)	Bakker et al. (2003)	Christou and Aristidou (2017)	Frommel et al. (2017)	Lindal et al. (2018)	Vlahovic et al. (2018)	Langbehn et al. (2018)	Coomer et al. (2018)	Griffin et al. (2018)	Clifton and Palmisano (2019)	Bozgeyikli et al. (2019)	Loup and Loup-Escande (2019)	Paris et al. (2019)	Boletsis and Cedergren (2019)	Drogemuller et al. (2020)	Kelly et al. (2020)	Cherep et al. (2020)
Teleportation Type																	
Operation	H	C	C	C	H	C	C	C	C	C	H	C	C	C	C	C	C
Translation	C+D	D	D	D	D	D	D	D	D	D	D	D	D	D	D	D	D
Range	Fx	Fr	Fr	Fx	Fr	Fr	Fr	Fr	Fr	Fr	Fr	Fr	Fr	Fr	Fr	Fr	Fr
Concordance	PC	PC + D	PC	PC	PC	PC	PC	PC	PC	PC	PC	PC	PC	PC	PC	PC + D	PC + D
Study																	
N^*o*^ of participants	10	20	18	24	40	29	33	10	20	25	15	44	16	26	25	24	24
Task	S	S	S	N	N	N	N	S	N + S	N	N	N + S	T	N	S	T	T
Comparison																	
Joystick		×		×		×*	×	×			×			×			
Walking																×	×
Walk in Place									×		×			×			
Redirected							×				×						
Walking																	
World in Miniature															×		
Arm Swinging												×					
Arm cycling								×									
Head Gaze			×														
Fixed Path				×	×												
Point tugging			×					×					×				
Steering										×							
Body/Head Tilt						×			×								
Hand Flapping											×						
Skiing													×				
Flying											×				×**		
Magic Carpet													×				
Stepper											×						
Machine																	
Trackball											×						
Significant Effects																	
VR Sickness			✓	✓	✓***		✓	✓	✓	✓	×	✓	×				
Performance			✓				✓	✓	✓		✓	×		✓	✓		
Presence				✓		×	×	✓	✓	✓	✓		×	✓			
Disorientation	✓	✓					✓						✓			✓	✓
Usability						✓	×	✓	✓		✓	✓		✓			
Preference							✓				✓				✓		

The teleportation operation types are: handsfree (H) and controller (C); the translation types are: continuous (C) and discontinuous (D); range types are: fixed (Fx) and free (Fr); and the concordance types are: partially concordant (PC) and discordant (D). The study task types are: navigation (N), triangle completion (T), and search (S). For significant effects, A ✓ indicates a significant effect, whereas a × means this factor was evaluated but no significant effect was found. An absence of either means this factor was not studied.

(*) Two variants of joystick locomotion, regular and one with the field of view restricted, were compared against.

(**) Two variants of flying, one handed flying and two handed flying were compared.

(***) Interaction effect of locomotion mode and exposure time was reported.

**TABLE 2 |:** (*) Funk et al. (2019) compared two variants of curved trajectory teleport to three point and teleport variants (straight trajectory, parabolic trajectory and parabolic trajectory with angle select). (**) Von Willich et al. (2020) didn’t find statistically significant improvement over point and teleport but got generally favorable user feedback. (***) Weissker et al. (2019) compared against a natural extension of regular teleport where the whole group teleports to a new position when the group navigator uses teleport.

	Bolte et al. (2011)	Bozgeyikli et al. (2016)	Linn (2017)	Bhandari et al. (2018b)	Liu et al. (2018)	Cmentowski et al. (2019)	Griffin and Folmer (2019)	Funk et al. (2019)	Von Willich et al. (2020)	Weissker et al. (2019)
New feature										
	Jumping based teleport	Post teleport rotation	Gaze based teleport	Optical flow/path integration	Optimal tracking space utilization	Continuous avatar representation	Continuous avatar representation	Curved teleport trajectory	Foot controlled teleport	Multi user teleport
Teleportation Type										
Operation	H	H	H/C	C	C	C	C	C	H	C
Translation	D	D	D	C	D	C	C	C	D	D
Range	Fr	Fr	Fr	Fr	Fr	Fr	Fr	Fr	Fr	Fr
Concordance	PC	D	PC	PC	D	D	D	PC	PC	PC
Study										
#participants	9	15	12	16	14	30	22	20	20	40
Task	N	N	N	T	N	N	N	N	N	
Comparison										
Regular	X		X	X	X	X	X	X*	X**	X*
Teleport										
Real walking	X									
Joystick		X								
Walk in Place		X								
Improvement										
Performance		X			X		X			X
Presence					X					
Disorientation	X			X		X				X
Usability	X							X		
Hands Free			X						X	
